# Comparing Triple Combination Drug Therapy and Traditional Monotherapy for Better Survival in Patients With High-Risk Hypertension: A Systematic Review

**DOI:** 10.7759/cureus.41398

**Published:** 2023-07-05

**Authors:** Mustafa Abrar Zaman, Nimra Awais, Travis Satnarine, Areeg Ahmed, Ayesha Haq, Deepkumar Patel, Sai Dheeraj Gutlapalli, Grethel N Hernandez, Kofi Seffah, Safeera Khan

**Affiliations:** 1 Internal Medicine, California Institute of Behavioral Neurosciences & Psychology, Fairfield, USA; 2 Internal Medicine, St. George's University School of Medicine, Newcastle upon Tyne, GBR; 3 Research, California Institute of Behavioral Neurosciences & Psychology, Fairfield, USA; 4 Pediatrics, California Institute of Behavioral Neurosciences & Psychology, Fairfield, USA; 5 Internal Medicine, California Institute of Neuroscience, Thousand Oaks, USA; 6 Family Medicine, California Institute of Behavioral Neurosciences & Psychology, Fairfield, USA; 7 Internal Medicine, Richmond University Medical Center Affiliated With Mount Sinai Health System and Icahn School of Medicine at Mount Sinai, New York, USA; 8 Internal Medicine Clinical Research, California Institute of Behavioral Neurosciences & Psychology, Fairfield, USA; 9 Internal Medicine, Piedmont Athens Regional Medical Center, Athens, USA

**Keywords:** awareness building, drug therapeutics, heart disease, high blood pressure, hypertension

## Abstract

High-risk hypertension patients are more susceptible to cardiovascular disease, stroke, and mortality. Monotherapy and triple combination drug therapy are two different approaches to treating hypertension. Monotherapy involves using a single medication to manage hypertension, whereas triple combination therapy involves the simultaneous use of three different antihypertensive medications from different drug classes. Making a fast switch from monotherapy to combination medication is one method to regulate blood pressure (BP) better. It is widely recognized that a significant proportion of individuals with hypertension require combination therapy to manage their condition effectively. This review aims to evaluate the mortality rates across monotherapy and triple combination drug therapy in high-risk hypertension patients. A systematic literature review was conducted across multiple scientific literature repositories. The review followed the Preferred Reporting Items for Systematic Reviews and Meta-Analyses (PRISMA) 2020 guidelines for systematic reviews and meta-analyses. Based on the end outcome of each published journal on the effectiveness of triple combination drug therapy as a treatment option for high-risk hypertension patients, there was a notable difference in overall survival, mortality rates, BP reduction, and adherence datasets. Triple combination drug use correlated with increased timeframes for multiple patient survival parameters within the articles shortlisted in this investigation. However, it is crucial for healthcare providers to weigh the risks and benefits of triple combination drug therapy when deciding which treatment approach is best for their patients.

## Introduction and background

Hypertension is a major public health concern, affecting millions of people worldwide [1]. The greatest population-based risk for cardiovascular disease is associated with hypertension, the most common cardiovascular risk factor [1,2]. Improved hypertension management is one of the most powerful public health and population healthcare levers for reducing years of life lost and disability-adjusted life years [2,3]. Sadly, hypertension and related cardiovascular and renal diseases are spreading globally. In the United States, according to data from the National Health and Nutrition Examination Survey (NHANES), between 2011 and 2014, only 53% of adults with hypertension had it under control, and the situation is even more concerning in other nations [4,5]. Individuals who have diabetes, chronic renal disease, stroke, established coronary artery disease, or a coronary artery disease equivalent are considered at increased risk of hypertension [6,7].

Nearly 75% of hypertensive patients require combination medication because they cannot control their blood pressure (BP) adequately with monotherapy. Making a fast switch from monotherapy to combination medication is one method to regulate BP better [8]. A target BP of 140/90 mmHg is advised by the Seventh Report of the Joint National Committee on Prevention, Detection, Evaluation, and Treatment of High Blood Pressure (JNC) [7], as well as recommendations made by the European Society of Hypertension (ESH) and the European Society of Cardiology (ESC) [9]. Nevertheless, despite the abundance of antihypertensive medications on the market, fewer than 50% of hypertensive patients have their BP under control [10]. Another report in the Journal of the American Medical Association shows that most hypertension patients have uncontrolled BP despite the widespread availability of effective antihypertensive medications [3]. In most nations, at least 50% of hypertensive patients do not meet the BP objectives that are generally advised [4,7,11]. 

Many patients will need three antihypertensive medications to reach BP targets, and current recommendations suggest combining medications with complementary modes of action. Three single-pill triple combination medications are on the market, each combining a calcium channel blocker, a diuretic, and an agent that affects the renin-angiotensin-aldosterone pathway (either a direct renin inhibitor or an angiotensin II receptor blocker) [7,8]. These triple combination medicines consistently showed a higher BP decrease than conventional monotherapy [7,12].

It is widely acknowledged that most hypertensive patients need combination medication. It has been anticipated that at least 25% of patients will need triple combination therapy to achieve BP control based on cumulative data from clinical studies [11,13,14]. As seen by the higher percentage of patients on triple combination medication attaining BP control after just two weeks at the maximum dose, such triple combination therapies have proven more effective than component dual combinations [15-18].

The aim of this systematic review is to thoroughly examine the array of research that already exists on the causes, identification, and management of hypertension. A considerable section of the world's population suffers from hypertension, also referred to as high BP, which is a chronic medical condition linked to a number of cardiovascular issues. The clinical and epidemiological papers released within the last 10 years will be the main focus of the study, according to the experts. The review seeks to offer an updated understanding of hypertension based on recent evidence by restricting the scope to recent publications. The review will take into account pertinent guidelines and evidence-based recommendations on managing hypertension in addition to original research papers. This review's main objective is to provide a summary of the most recent research on hypertension, including information on its etiology (causes), diagnosis, and treatment options. The results of this systematic review will be helpful for academics, policymakers, and healthcare professionals working on the subject of hypertension. The review presents an up-to-date and thorough assessment of the current body of information, points out areas in which more study is necessary, and may direct the creation of new research and treatment recommendations. By ensuring that healthcare practices are in line with the most comprehensive and recent evidence available, this systematic review hopes to improve patient treatment and progress the study of hypertension.

## Review

Method

The systematic review was conducted using the Preferred Reporting Items for Systematic Reviews and Meta-Analyses (PRISMA) 2020 [12]. 

Search Strategy and Data Extraction 

We used the medical databases PubMed, MEDLINE, PubMed Central (PMC), ResearchGate, ScienceDirect, Google Scholar, and Cochrane Library to search for relevant full-text and peer-reviewed articles. The databases were searched using the predetermined keywords to find potentially relevant articles. The keywords used in the search included Hypertension, Triple Combination Drug Therapy, High-Risk Patients, Blood Pressure Control, and Monotherapy. Keywords were used with the Boolean "AND" and "OR" to obtain results. Following Medical Subject Heading (MeSH) search strategy was also used in PubMed MeSH database: “High”[All Fields] AND (“risk”[MeSH Terms] OR “risk”[All Fields]) AND (“patient s”[All Fields] OR “patients”[MeSH Terms] OR “patients”[All Fields] OR “patient”[All Fields] OR “patients s”[All Fields]) AND (“hypertense”[All Fields] OR “hypertension”[MeSH Terms] OR “hypertension”[All Fields] OR “hypertension s”[All Fields] OR “hypertensions”[All Fields] OR “hypertensive”[All Fields] OR “hypertensive s"[All Fields] OR "hypertensives"[All Fields]) AND (("triple"[All Fields] OR "triple“"[All Fields]) AND (“drug therapy, combination”[MeSH Terms] OR (“drug”[All Fields] AND “therapy”[All Fields] AND “combination”[All Fields]) OR “combination drug therapy”[All Fields] OR (“combination”[All Fields] AND “drug”[All Fields] AND “therapy”[All Fields]))) AND (“monotherapies”[All Fields] OR “monotherapy”[All Fields]) AND ((“blood pressure”[MeSH Terms] OR (“blood”[All Fields] AND “pressure”[All Fields]) OR “blood pressure”[All Fields] OR “blood pressure determination”[MeSH Terms] OR (“blood”[All Fields] AND “pressure”[All Fields] AND “determination”[All Fields]) OR “blood pressure determination”[All Fields] OR (“blood”[All Fields] AND “pressure”[All Fields]) OR “blood pressure”[All Fields] OR “arterial pressure”[MeSH Terms] OR (“arterial”[All Fields] AND “pressure”[All Fields]) OR “arterial pressure”[All Fields] OR (“blood”[All Fields] AND “pressure”[All Fields])) AND (“controlling”[All Fields] OR “controllability”[All Fields] OR “controllable”[All Fields] OR “controllably”[All Fields] OR “controller”[All Fields] OR “controller s”[All Fields] OR “controllers”[All Fields] OR “controlling”[All Fields] OR “controls”[All Fields] OR “prevention and control”[MeSH Subheading] OR (“prevention”[All Fields] AND “control”[All Fields]) OR “prevention and control”[All Fields] OR “control”[All Fields] OR “control groups”[MeSH Terms] OR (“control”[All Fields] AND “groups”[All Fields]) OR “control groups”[All Fields])).

Eligibility and Screening

High-risk hypertension refers to a condition where individuals with hypertension are at a higher risk of experiencing complications or adverse health outcomes. This category is defined by the following criteria: systolic BP (SBP) ≥180 mmHg and/or diastolic BP (DBP) ≥110 mmHg. The SBP represents the top number in a BP reading, indicating the pressure in the arteries when the heart beats. An SBP reading of 180 mmHg or higher falls within the high-risk range. DBP is the lower number that shows the pressure in the arteries when the heart is resting between beats. If the DBP is 110 mmHg or more, it is considered high risk [14,17]. Low-risk hypertension is defined as no atherosclerotic cardiovascular disease (ASCD) or 10-year cardiovascular disease (CVD) risk <10% with stage 1 hypertension (BP 130-139/80-89 mmHg). Low-risk hypertension is when individuals with hypertension have a lower risk of complications than those in the high-risk category. The following criteria define this category: no ASCD. ASCD refers to the buildup of plaque in the arteries, which can lead to various cardiovascular problems. If an individual with hypertension has no pre-existing ASCD, they are classified as low risk. A 10-year CVD risk <10% with stage 1 hypertension implies that the individual has a cardiovascular disease risk of less than 10% over a span of 10 years. If an individual with stage 1 hypertension (BP ranging from 130-139 mmHg systolic and/or 80-89 mmHg diastolic) has a CVD risk of less than 10%, they are considered low risk [16,18].

Table 1 shows the inclusion and exclusion criteria for the systematic review.

**Table 1:** Inclusion and exclusion criteria employed for this study.

Inclusion criteria	Exclusion criteria
Papers published within 10 years	Gray literature
Papers written and published in the English language	Papers involving low-risk hypertension patients who underwent monotherapy
Papers involving human subjects	Papers involving low-risk hypertension patients who underwent triple combination drug therapy
Papers involving both genders	Papers involving animal subjects
Papers focusing on all age groups	Papers with only abstract available
Papers focused on triple combination drug therapy for patients with high-risk hypertension	
Papers focused on monotherapy for patients with high-risk hypertension	

The selected articles were subjected to quality assessment using the relevant techniques. To assess randomized controlled trials (RCTs), the Cochrane Bias Assessment Tool was used, and to assess observational studies, the Newcastle-Ottawa Tool was used [19,20]. For systematic reviews, the Assessment of Multiple Systematic Reviews (AMSTAR) Checklist was used [21]. Table 2 shows the quality appraisal tools used to assess bias in the studies.

**Table 2:** Quality appraisal tools employed for this study. AMSTAR: Assessment of Multiple Systematic Reviews; RCT, randomized controlled trials.

Study type	Quality appraisal tool
RCT	Cochrane Bias Assessment Tool
Systematic reviews	AMSTAR Checklist
Observational studies	Newcastle-Ottawa Tool

Results

**Figure 1:**
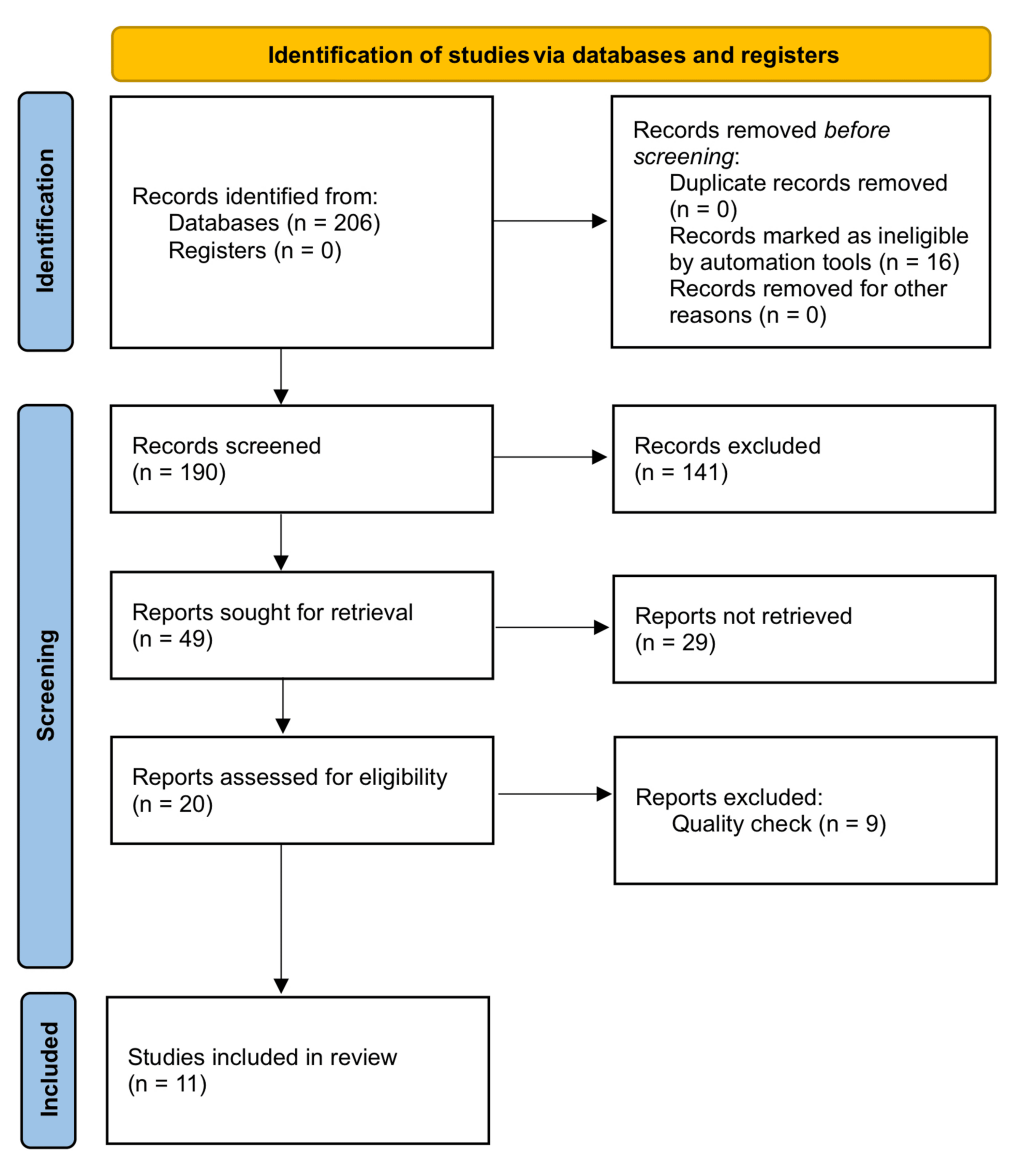
PRISMA 2020 PRISMA: Preferred Reporting Items for Systematic Reviews and Meta-Analyses.

We searched databases from Medline, PubMed, PMC, ScienceDirect, Google Scholar, and Cochrane Library for 206 published papers. There were 50 articles after the duplicates were eliminated, and the articles were filtered based on the inclusion and exclusion criteria. Only 11 pertinent journal articles were left after the screening and eligibility. There were two observational studies [3,8], six systematic reviews [1,5,7,9,11,14], and three RCTs [6,10,13]. Figure 1 shows the summary of the PRISMA flow chart.

Based on the selection analysis of 11 published journal articles, there were a total of approximately 777,264 patients included in the studies. Table 3 presents a summary of the reviewed studies.

**Table 3:** Publication summary of the purpose, number of patients, study type, and conclusion. A+B+C: angiotensin-converting inhibitor + beta-blocker + calcium channel blocker; A+C+D: angiotensin-converting inhibitor + calcium channel blocker + diuretics; BP: blood pressure; FDTC: Family Drug Treatment Court; MACE: major adverse cardiovascular events; n: number; PAINT: Perindopril-Amlodipine plus Indapamide combination for controlled hypertension Non-intervention Trial; RAAS: renin-angiotensin-aldosterone system; RCT: randomized controlled trial; RH: resistant hypertension; SPC: single-pill combination; SR: sustained release; SBP: systolic blood pressure; DBP: diastolic blood pressure.

Author and year of publication	Purpose	Number of patients	Study type	Results
Egan et al., 2022 [1]	This article provides a detailed overview and summary of the current literature regarding the use of SPCs for managing hypertension, with a particular focus on their use as initial therapy compared to monotherapy and multiple pill regimens. The review examines the impact of SPC on adherence, hypertension control, clinical outcomes, population health, and adverse effects. Additionally, an attempt is made to quantify the relative use of SPC compared to monotherapy and free-dose combinations in hypertension management. The barriers and potential pathways to more effective implementation and use of SPC in managing hypertension are also explored.	>250,000	Systematic Review	Research findings indicate that the incorporation of antihypertensive SPCs in national formularies, the endorsement of SPC utilization in national hypertension guidelines, and the convenient accessibility and cost-effectiveness of SPC in the market enhance the adoption of this therapeutic alternative.
Páll et al., 2014 [2]	The PAINT study was primarily designed to investigate the antihypertensive efficacy of a triple combination of antihypertensive drugs, namely perindopril, amlodipine, and indapamide SR, in patients who had previously been treated for hypertension but had not achieved target BP values. As a secondary objective, the study also analyzed changes in metabolic parameters.	6,088	Observational Study	The administration of a triple combination comprising perindopril, amlodipine, and indapamide SR has demonstrated effective control of BP in hypertensive patients who have previously experienced uncontrolled BP while under treatment with either a single antihypertensive drug or a combination therapy that included a RAAS inhibitor and amlodipine or hydrochlorothiazide.
Düsing et al., 2017 [4]	It has been estimated that a significant portion of patients require the use of three or more antihypertensive agents. The combination of agents from different classes has resulted in a notable reduction in BP, approximately five times greater than the effect of doubling the dose of a single agent. Furthermore, the use of drugs with complementary mechanisms of action may offer additional benefits beyond BP reduction, such as improved tolerability and higher rates of adherence to prescribed medication, in comparison with increasing the dose of a single agent.	>500,223	Systematic Review	In conclusion, combination therapy, including drugs from classes having complementary action, is advantageous in terms of BP reduction and control, particularly in high-risk patients, and may be associated with improved tolerability. Administration of combination therapy as SPC is capable of enhancing treatment adherence.
Lin et al., 2015 [5]	RH is strongly correlated with cardiovascular risks. The limitation of enrolling a large number of study participants constrains research on the treatment effects of RH. This research aims to assess the efficiency of treating resistant hypertension in Taiwanese patients using a triple combination therapy of antihypertensive drugs.	13,551	RCT	The A+C+D combination therapy seemed more effective than the A+B+C therapy in preventing MACE among patients with RH.
Volpe et al., 2020 [6]	As aforementioned, one-fourth to one-third of hypertensive patients fail to achieve BP control with dual combination therapies, requiring three or more antihypertensive agents.	>2,271	Systematic Review	Many hypertensive patients do not achieve adequate BP control with monotherapy or dual therapy, with almost one-third of subjects requiring three or more drugs to reach therapeutic targets. Triple combination therapy has been demonstrated to provide better results regarding the percentage of well-controlled subjects, office and 24-hour BP reduction, and time to achieve BP goals compared to dual-combination therapy, without a significant increase in adverse effects events.
Mazza et al., 2017 [7]	The study aimed to determine whether the FDTC or free combination therapy is more effective and easier to tolerate in reducing office and 24-hour BP levels.	92	Observational Study	The study finds administration of fixed-dose triple combination therapy comprising perindopril/indapamide/amlodipine has effectively reduced systolic and pulse pressure levels among patients exhibiting moderate hypertension that remains uncontrolled despite the use of dual fixed combination therapy.
Gorostidi and de la Sierra, 2013 [8]	The review aims to elucidate the benefits of combination therapy in managing hypertension, as well as the effectiveness of various antihypertensive combinations.	>3,666	Systematic Review	For the majority of patients with hypertension, the combination of antihypertensive drugs may be essential to attain satisfactory BP goals. Approaches to enhance BP control include the rapid transition from monotherapy to combination therapy, the primary treatment with a two-drug combination, and the application of fixed-dose combinations in a single pill.
Salam et al., 2014 [9]	The proposed study is a prospective, open, randomized, controlled clinical trial with a sample size of 700. It aims to compare the triple pill–based strategy to usual care in individuals with persistent mild-to-moderate hypertension. The inclusion criteria for the study are SBP of 140 mm Hg and DBP of 90 mm Hg, or SBP of 130 mm Hg and DBP of 80 mm Hg in patients with diabetes or chronic kidney disease, who are not on any or minimal drug therapy.	700	RCT	The present study aims to evaluate the potential benefits of early utilization of a low-dose triple combination therapy in tackling the challenges associated with hypertension control. The study seeks to investigate if such a strategy can aid in achieving BP control earlier, ensuring better adherence, and reducing the occurrence of adverse effects during less intensive clinical follow-up.
Epstein et al., 2013 [10]	The purpose of this review is to assess the efficacy and potential benefits of implementing fixed-dose triple combination therapy for the treatment and control of hypertension.	>407	Systematic Review	The utilization of combination therapy is a widely accepted cornerstone in the management of hypertension. It has been estimated that approximately one in every four patients will necessitate the use of three antihypertensive agents to attain proper BP control.
Konradi et al., 2022 [12]	The present study aims to assess the effectiveness and safety of the triple therapy comprising amlodipine 5 mg, bisoprolol 5 mg, and perindopril 5 mg in both SPC and other combination formats over a period of 12 weeks.	150	RCT	When faced with uncontrolled BP despite monotherapy at the maximum dose or bi-therapy at the initial dose, it may be beneficial to switch to triple therapy of amlodipine 5 mg, bisoprolol 5 mg, and perindopril 5 mg. This approach has resulted in a significant decrease in BP (p < 0.001), improved response to treatment, and a better BP control rate.
Thomopoulos et al., 2019 [13]	A systematic review and meta-analysis of RCTs comparing double-drug and triple-drug BP-lowering combinations support the guideline view that triple-drug combinations reduce BP levels to a greater extent than double-drug combinations.	116	Systematic Review	Until then, triple-drug combination antihypertensive treatments should replace dual-drug combinations whenever patients remain uncontrolled

Discussion

Traditional Monotherapy for High-Risk Hypertension

Hypertension, commonly known as high BP, is the most prevalent cardiovascular risk factor and has been extensively researched. Despite the availability of various treatment options, studies have shown that only 40-50% of treated hypertensive patients are able to achieve the recommended treatment objectives [7]. This indicates a significant gap between the desired BP levels and the actual control achieved in clinical practice. The introduction of more aggressive therapeutic targets in recent guidelines, such as the 2018 ESC/ESH and the 2017 American Heart Association/American College of Cardiology (AHA/ACC) guidelines, further emphasizes the importance of achieving optimal BP control. These guidelines recommend lower SBP levels between 130 and 120 mmHg and DBP levels between 80 and 70 mmHg for most high-risk hypertensive patients.

Traditional monotherapy, involving the use of a single antihypertensive drug, has long been considered the cornerstone of hypertension treatment due to its cost-effectiveness. Several classes of antihypertensive drugs, including angiotensin-converting enzyme (ACE) inhibitors, angiotensin receptor blockers (ARBs), calcium channel blockers, diuretics, and beta blockers, are commonly used as monotherapy options [5]. However, despite the widespread use of monotherapy, the proportion of uncontrolled hypertensive patients remains substantial. The discrepancy between the recommended treatment objectives and the actual control achieved suggests the need for reevaluating the current treatment strategies and exploring alternative approaches to improve BP management.

One potential explanation for the suboptimal control in treated hypertensive patients could be related to the complexity of hypertension itself. It is increasingly recognized that hypertension is a multifactorial condition influenced by various physiological and genetic factors. The heterogeneity of hypertension and individual patient characteristics may contribute to the variable treatment response observed in clinical practice [7]. Another factor that might impact the effectiveness of monotherapy is the presence of comorbidities in hypertensive patients. Many individuals with hypertension often have other underlying health conditions, such as diabetes, dyslipidemia, or renal dysfunction, which can complicate management and require a more tailored treatment approach. Comorbidities may necessitate the use of multiple medications or combination therapies to achieve optimal BP control [6]. Furthermore, patient adherence to prescribed antihypertensive medications plays a crucial role in determining treatment success. Poor adherence to medication regimens is a known challenge in the management of chronic conditions, including hypertension [5]. Non-adherence can result from various factors, such as the complexity of medication regimens, side effects, cost, lack of understanding about the importance of treatment, or simply forgetfulness. Strategies to enhance patient education and promote medication adherence should be incorporated into clinical practice to improve treatment outcomes.

Despite the extensive research and availability of various antihypertensive medications, a significant proportion of treated hypertensive patients fail to achieve the recommended treatment objectives. The introduction of more aggressive therapeutic targets in recent guidelines further emphasizes the need for improved BP control. While traditional monotherapy has been the mainstay of hypertension treatment, alternative approaches, such as combination therapy or personalized treatment strategies, may be necessary to address the complexity and heterogeneity of hypertension [7]. Additionally, efforts to enhance patient adherence to medication regimens and optimize patient education should be prioritized to improve treatment outcomes in hypertensive individuals.

Benefits and Limitations of Traditional Monotherapy

Traditional monotherapy involves the use of a single antihypertensive drug to regulate BP. The key advantage of this approach is its cost-effectiveness, as only one medication is required [10]. Additionally, traditional monotherapy is relatively easy to administer and supervise, making it a convenient option for patients. However, despite these advantages, traditional monotherapy has several limitations that need to be considered. One significant limitation is its potential inadequacy in regulating BP for individuals with complex medical conditions requiring multiple medications. Hypertension is often a multifactorial condition, and some patients may require a combination of drugs to effectively control their BP. Using a single antihypertensive drug may not provide sufficient control in these cases [1]. This limitation is particularly relevant for patients with high-risk hypertension, as they often need aggressive BP management to reduce the risk of cardiovascular complications.

Another limitation of traditional monotherapy is its limited efficacy in achieving BP control, even at the highest prescribed doses. Despite escalating the dosage, many patients with high-risk hypertension do not achieve the desired BP targets with monotherapy alone. This suggests that monotherapy may not be sufficient for all patients, especially those with more severe forms of hypertension [1].

Relying solely on monotherapy for BP control may increase the risk of side effects. Some patients may require higher doses of a single medication to achieve adequate BP control, which can increase the likelihood of adverse reactions and complications associated with the drug [3]. Additionally, certain patients may experience side effects from a specific antihypertensive drug, making it necessary to explore alternative treatment options that are better tolerated. The limitations of traditional monotherapy highlight the need for a more comprehensive approach to hypertension management. Combination therapy, which involves the use of multiple antihypertensive drugs with different mechanisms of action, has emerged as an alternative strategy that addresses some of the shortcomings of monotherapy. Combination therapy allows for synergistic effects and improved BP control, making it a valuable option for patients who do not respond adequately to monotherapy alone [1].

Traditional monotherapy offers cost-effectiveness and ease of administration, but it may not provide sufficient BP control for patients with complex medical conditions or high-risk hypertension. The limitations of monotherapy, including its limited efficacy and increased risk of side effects, necessitate the exploration of alternative treatment strategies such as combination therapy [7]. Future research should focus on identifying patient subgroups that would benefit most from combination therapy and optimizing treatment regimens to achieve better BP control and minimize adverse events.

Triple Combination Drug Therapy for High-Risk Hypertension

The management of high-risk hypertension has been a significant challenge in clinical practice, often requiring multiple medications to achieve optimal BP control. The traditional approaches of tailored therapy and stepped care have demonstrated limitations in providing adequate BP control and have proven to be expensive and time-consuming [6]. In recent years, triple combination drug therapy has emerged as a promising strategy for managing high-risk hypertension, offering a more efficient and effective alternative to standard care [8]. Triple combination drug therapy involves the simultaneous administration of three different antihypertensive medications to lower BP. This approach has gained popularity due to its ability to achieve target BP levels in patients who are unable to reach their goals with monotherapy or require multiple medications [6]. The findings from our study support the notion that triple combination therapy is more effective and acceptable than standard care for managing hypertension [10].

One of the notable advantages of triple combination therapy is its superior efficacy in achieving BP control. Initiating antihypertensive treatment with a pharmacological combination and maintaining it have been associated with a significant reduction in cardiovascular risk compared to starting with monotherapy and later transitioning to a combination or returning to a single drug after initial combination use [7]. This observation highlights the importance of early initiation and sustained use of triple combination therapy in high-risk hypertensive patients. Triple combination therapy offers several potential benefits over traditional treatment approaches. Firstly, it simplifies medication regimens for patients, reducing pill burden and potentially improving treatment adherence. By combining three antihypertensive agents with complementary mechanisms of action, triple combination therapy addresses multiple pathways involved in BP regulation, enhancing its overall efficacy [8]. This approach also provides a more comprehensive approach to hypertension management, allowing for a more tailored and individualized treatment strategy. While triple combination therapy has demonstrated promising results, there are some considerations that should be acknowledged [10]. The use of multiple medications increases the potential for drug interactions and adverse effects, necessitating careful monitoring and appropriate dose adjustments. Healthcare professionals need to be mindful of the possible pharmacokinetic and pharmacodynamic interactions between the chosen antihypertensive agents when designing a triple combination regimen.

Additionally, the cost implications of triple combination therapy should be taken into account. The use of multiple medications may increase the financial burden on patients, especially in regions where healthcare costs are not fully covered by insurance or public healthcare systems. Future research should focus on evaluating the cost-effectiveness of triple combination therapy and assessing its long-term economic impact [7]. Triple combination drug therapy represents a promising approach for managing high-risk hypertension. This strategy has demonstrated superior efficacy and acceptability compared to traditional approaches, providing a valuable option for patients who are unable to achieve adequate BP control with monotherapy or require multiple medications [1].

Benefits and Limitations of Triple Combination Drug Therapy

Triple combination drug therapy has emerged as an effective approach in the management of hypertension, particularly in patients who are unable to achieve adequate BP control with monotherapy alone. This therapy offers several benefits, but it also comes with certain limitations that need to be considered. 

One of the main advantages of triple medication combination therapy is its superior efficacy compared to conventional monotherapy. Numerous studies have demonstrated that the use of three antihypertensive medications in combination leads to a greater reduction in BP levels [10]. This finding is particularly relevant for patients who have failed to achieve target BP goals with monotherapy. By utilizing multiple medications with different mechanisms of action, triple therapy can effectively target different pathways involved in BP regulation, resulting in better control.

However, despite its efficacy, triple medication combination therapy presents several limitations. One notable concern is the increased complexity and cost associated with administering and monitoring multiple medications simultaneously. The need for patients to take multiple medications can lead to higher treatment costs and may pose challenges in terms of adherence. This is particularly relevant for elderly and frail individuals who may have difficulty managing complex medication regimens [1]. These patients often require additional support and monitoring to ensure adherence and minimize the risk of adverse events. Another important consideration is the potential difficulty in identifying the specific medication responsible for adverse effects when multiple pharmaceutical agents are used in combination. Adverse effects can occur with any medication, and the presence of multiple drugs in a triple combination regimen can complicate the identification of the causative agent. This challenge may hinder the ability to make adjustments or substitutions to optimize therapy and minimize side effects. 

Moreover, the use of triple combination drug therapy increases the likelihood of experiencing side effects [11]. Each additional medication introduces a new set of potential adverse events, and the cumulative effect of these drugs may pose a greater risk to patients. It is crucial for healthcare providers to closely monitor patients on triple therapy and promptly address any adverse effects that arise. Patient adherence is a critical factor in the success of any antihypertensive treatment regimen. Poor adherence can compromise BP control and lead to suboptimal outcomes. Many different things can cause poor adherence. In a poll of 1432 hypertension patients, those who had difficulties remembering to take their antihypertensive medicine (n = 407) cited the following as the primary causes: cost (32.4%), side effects (12.5%), and lack of insurance (22.4%) [12]. These findings emphasize the need for a comprehensive approach to patient care, taking into account not only the selection of appropriate medications but also the consideration of factors that may affect adherence.

Triple combination drug therapy offers an effective strategy for achieving BP control in patients who are unresponsive to monotherapy. It provides superior efficacy compared to conventional approaches, but it is not without limitations. The complexity and cost of administering and monitoring multiple medications, the challenge of identifying the causative agent of adverse effects, the increased risk of side effects, and poor adherence are important considerations. Future studies should continue to explore the optimal selection and combination of antihypertensive agents, assess long-term outcomes and cost-effectiveness, and further investigate the potential benefits and risks associated with triple combination therapy.

Better Survival With Triple Combination Drug Therapy

Several studies have shown that triple combination drug therapy is associated with improved survival in patients with high-risk hypertension [6,9]. Most of the evidence comes from the studies testing valsartan/amlodipine/hydrochlorothiazide and olmesartan/amlodipine/hydrochlorothiazide combinations. Two large, randomized, double-blind, controlled trials have shown that these two triple combinations promoted BP reductions, thus leading to lower mortality rates [9]. In case of uncontrolled BP despite monotherapy at maximal dose or bi-therapy at initial dose, switching to triple therapy of amlodipine 5 mg+bisoprolol 5 mg+perindopril 5 mg leads to a significant decrease in BP (p < 0.001), an improvement in response to treatment, and a better BP control rate with a high survival rate [13].

This review study has limitations, mostly due to the exclusion of particular meta-analyses, such as the use of the random-effects model, estimation of impact sizes, estimation of sensitivity for heterogeneity sub-groups, and little investigation effects [6,14]. Furthermore, this review did not consider new evidence about triple combination therapy as only publications with full-text free of charge were used in this review; paid articles were not included [15].

## Conclusions

Triple combination medicine and conventional monotherapy can impact the survival rate of patients with high-risk hypertension. Compared to conventional monotherapy, the administration of the triple combination medicine was associated with a significantly lower risk of death, according to the study's findings. The triple combination medication lowered the risk of heart failure, stroke, and other cardiovascular events. This shows that people with high-risk hypertension may benefit from a triple combination medication. While this approach is more expensive and complex than traditional monotherapy, it may be beneficial for patients who cannot achieve their target BP with monotherapy or require multiple medications. Healthcare providers need to weigh the risks and benefits of triple combination therapy when deciding which treatment approach is best for their patients.

## References

[1] Egan BM, Kjeldsen SE, Narkiewicz K, Kreutz R, Burnier M (2022). Single-pill combinations, hypertension control and clinical outcomes: potential, pitfalls and solutions. Blood Press.

[2] Páll D, Szántó I, Szabó Z (2014). Triple combination therapy in hypertension: the antihypertensive efficacy of treatment with perindopril, amlodipine, and indapamide SR. Clin Drug Investig.

[3] Chow CK, Teo KK, Rangarajan S (2013). Prevalence, awareness, treatment, and control of hypertension in rural and urban communities in high-, middle-, and low-income countries. JAMA.

[4] Düsing R, Waeber B, Destro M, Santos Maia C, Brunel P (2017). Triple-combination therapy in the treatment of hypertension: a review of the evidence. J Hum Hypertens.

[5] Lin C, Tsai M, Chen W, Chuang P, Tang C (2015). Comparative effectiveness of triple antihypertensive combination therapy for patients with resistant hypertension in taiwan. Value Health.

[6] Volpe M, Gallo G, Tocci G (2020). New approach to blood pressure control: triple combination pill. Trends Cardiovasc Med.

[7] Mazza A, Lenti S, Schiavon L, Sacco AP, Dell'Avvocata F, Rigatelli G, Ramazzina E (2017). Fixed-dose triple combination of antihypertensive drugs improves blood pressure control: from clinical trials to clinical practice. Adv Ther.

[8] Gorostidi M, de la Sierra A (2013). Combination therapy in hypertension. Adv Ther.

[9] Salam A, Webster R, Singh K (2014). TRIple pill vs Usual care Management for Patients with mild-to-moderate Hypertension (TRIUMPH): study protocol. Am Heart J.

[10] Epstein BJ, Shah NK, Borja-Hart NL (2013). Management of hypertension with fixed-dose triple-combination treatments. Ther Adv Cardiovasc Dis.

[11] Haddaway NR, Page MJ, Pritchard CC, McGuinness LA (2022). PRISMA2020: an R package and Shiny app for producing PRISMA 2020-compliant flow diagrams, with interactivity for optimised digital transparency and Open Synthesis. Campbell Syst Rev.

[12] Konradi A, Hassan Y, Lefay D, Isachenko E, Jamois A (2022). Clinical efficacy and safety of amlodipine/bisoprolol/perindopril single-pill and free combination therapy in patients with uncontrolled essential hypertension. J Hypertens.

[13] Thomopoulos C, Michalopoulou H, Makris T (2019). Antihypertensive treatment escalation. J Hypertens.

[14] Tsai WC (2011). Treatment options for hypertension in high-risk patients. Vasc Health Risk Manag.

[15] Singh S (2017). How to conduct and interpret systematic reviews and meta-analyses. Clin Transl Gastroenterol.

[16] Goetsch MR, Tumarkin E, Blumenthal RS, Whelton SP. (2023). Goetsch MR, Tumarkin E, Blumenthal RS, Whelton SP. New Guidance on Blood Pressure Management in Low-Risk Adults With Stage 1 Hypertension. [online] American College of Cardiology. Am. Coll. Cardiol.

[17] Erdine S, Ro YM, Tse HF (2009). Single-pill amlodipine/atorvastatin helps patients of diverse ethnicity attain recommended goals for blood pressure and lipids (the Gemini-AALA study). J Hum Hypertens.

[18] Rossiyah N (2023). Nonalcoholic fatty liver disease and risk of incident hypertension: a systematic review. J. Adv. Med.

[19] Abou-Setta AM, Beaupre LA, Jones CA (2011). Newcastle-Ottawa Scale Assessment of Cohort Studies. https://www.ncbi.nlm.nih.gov/books/NBK56664/.

[20] (2023). Cochrane Methods Bias. Risk of Bias Tool. https://methods.cochrane.org/bias/risk-bias-tool.

[21] www.ncbi.nlm.nih.gov www.ncbi.nlm.nih.gov (2023). AMSTAR Checklist for the Quality Assessment of Systematic Reviews. https://www.ncbi.nlm.nih.gov/books/NBK263391/.

