# Correction: Comparing Triple Combination Drug Therapy and Traditional Monotherapy for Better Survival in Patients With High-Risk Hypertension: A Systematic Review

**DOI:** 10.7759/cureus.c274

**Published:** 2025-08-26

**Authors:** Mustafa Abrar Zaman, Nimra Awais, Travis Satnarine, Areeg Ahmed, Ayesha Haq, Deepkumar Patel, Sai Dheeraj Gutlapalli, Grethel N Hernandez, Kofi Seffah, Safeera Khan

**Affiliations:** 1 Internal Medicine, California Institute of Behavioral Neurosciences & Psychology, Fairfield, USA; 2 Internal Medicine, St. George's University School of Medicine, Newcastle upon Tyne, GBR; 3 Research, California Institute of Behavioral Neurosciences & Psychology, Fairfield, USA; 4 Pediatrics, California Institute of Behavioral Neurosciences & Psychology, Fairfield, USA; 5 Internal Medicine, California Institute of Neuroscience, Thousand Oaks, USA; 6 Family Medicine, California Institute of Behavioral Neurosciences & Psychology, Fairfield, USA; 7 Internal Medicine, Richmond University Medical Center Affiliated With Mount Sinai Health System and Icahn School of Medicine at Mount Sinai, New York, USA; 8 Internal Medicine Clinical Research, California Institute of Behavioral Neurosciences & Psychology, Fairfield, USA; 9 Internal Medicine, Piedmont Athens Regional Medical Center, Athens, USA

This article has been corrected to fix the following typos in the PRISMA diagram:

- "Reports sought for retrieval (n=**50**)" has been corrected to "Reports sought for retrieval (n=**49**)"
- "Reports assessed for eligibility (n=**21**)" has been corrected to "Reports assessed for eligibility (n=**20**)"
- "Reports excluded: Quality check (n=**10**)" has been corrected to "Reports excluded: Quality check (n=**9**)"

